# Retraction mechanics of Finochietto-style self-retaining thoracic retractors

**DOI:** 10.1186/s12938-019-0664-z

**Published:** 2019-04-16

**Authors:** Guillaume Chanoit, Charles A. Pell, Gil Bolotin, Gregory D. Buckner, Jeffrey P. Williams, Hugh C. Crenshaw

**Affiliations:** 1 Bristol Veterinary School and Bristol Heart Institute, University of Bristol, Langford, Bristol, BS40 5DU UK; 2 Physcient Inc., 112 South Duke St., Suite 4A, Durham, NC 27701 USA; 3 Cardiac Surgery Department, Rambam Health Care Campus, P.O.B. 9602, 31096 Haifa, Israel; 4 Department of Mechanical and Aerospace Engineering, North Carolina State University, Campus Box 7910, Raleigh, NC 27695 USA; 5 College of Veterinary Medicine, North Carolina State University, Raleigh, NC 27606 USA

**Keywords:** Retraction, Thoracotomy, Sternotomy, Finochietto, Rib fracture, Force relaxation

## Abstract

**Objectives:**

Analyze the mechanics of Finochietto-style retractors, including the responses of thoracic tissues during thoracotomy, with an emphasis on tissue trauma and means for its reduction.

**Methods:**

Mechanical analyses of the retractor were performed, including analysis of deformation under load and kinematics of the crank mechanism. Thoracotomies in a porcine model were performed in anesthetized animals (7) and fresh cadavers (17) using an instrumented retractor.

**Results:**

Mechanical analyses revealed that arm motion is a non-linear function of handle rotation, that deformation of the retractor under load concentrates force at one edge of the retractor blade, and that the retractor behaves like a spring, deforming under the load of retraction and continuing to force open the incision long after crank rotation stops. Experimental thoracotomies included retractions ranging from 50 to 112 mm over 30 to 370 s, generating maximum forces of 118 to 470 N (12–50 kgf). Tissue ruptures occurred in 12 of the 24 retractions. These ruptures all occurred at retraction distances wider than 30 mm and at forces greater than 122.5 N. Significant tissue ruptures were observed for nearly all retractions at higher retraction rates (exceeding ½ rotation of the crank per 10 s).

**Conclusions:**

The Finochietto-style retractor can generate large forces and some aspects of its design increase the probability of tissue trauma.

## Introduction

Thoracic retraction is used for surgical access via thoracotomy and sternotomy, with about 2 million open thoracic surgeries worldwide each year. Even when the intra-thoracic procedure is successful, trauma from retraction can cause complications, including rib fractures [1–5], impaired respiratory function [6–8], and pain, both acute and long-term [9–13]. This has led to extensive efforts to develop improvements (e.g. muscle sparing techniques [14–17], muscle flaps [18–20], and intracostal sutures [4, 21]) and, more broadly, minimally invasive alternatives (e.g., mini-thoracotomy/sternotomy and thoracoscopy).

The retractor developed by Enrique Finochietto in 1936 and published in 1941 [22] is widely used, and almost all other thoracic retractors (e.g., Ankeney, DeBakey, and Cooley) use Finochietto’s self-retaining ratchet (see Bonfils-Roberts [22] for a review). However, despite 75 years of widespread use, mechanical analyses of Finochietto-style retractors and biomechanical studies of thoracic retraction are virtually nonexistent in the literature, despite the trauma which it generates. It is widely accepted that slower retractions are less traumatic [4, 18, 22], and recently published sternotomy retraction results using dummies and human cadavers have demonstrated that forces applied to hemisternum can reach 350 N (Aigner et al. [23] and Saggio et al. [24]). Bolotin et al. [25–27], using a novel instrumented retractor, published measurements of forces during retraction in a sheep model and, importantly, demonstrated that force monitoring can reduce tissue trauma. Our goal was to analyze the mechanics of Finochietto-style thoracic retractors with an emphasis on the biomechanics of thoracic tissues, features of the retractor that increase tissue trauma, and provide guidelines for its use that can minimize trauma.

## Materials and methods

### Mechanical analysis

Kinematic analyses and static load deflection tests were performed on an instrumented Finochietto retractor (Fig. 1). Retraction force was measured using four strain gages (Vishay Micro-measurements model EA-06-125PC-350/LE) mounted in a full Wheatstone bridge configuration on each of the retractor blades (top center inside, bottom center inside, top center outside, and bottom center outside); gage outputs were routed to an AC signal conditioner/amplifier (Omega Engineering model DMD-465WB) and calibrated using applied dead weights. The distance between retractor arms at their bases (DA, Fig. 1) was measured using a linear displacement sensor (Transducers Direct model TD39056W). Both measurements were continuously monitored using a custom LabVIEW virtual instrument (National Instruments, Austin TX). The distance between retractor blades, DB, was measured using a modified draftsman’s caliper across the midpoints (between the proximal and distal edges) of each blade.

**Fig. 1:**
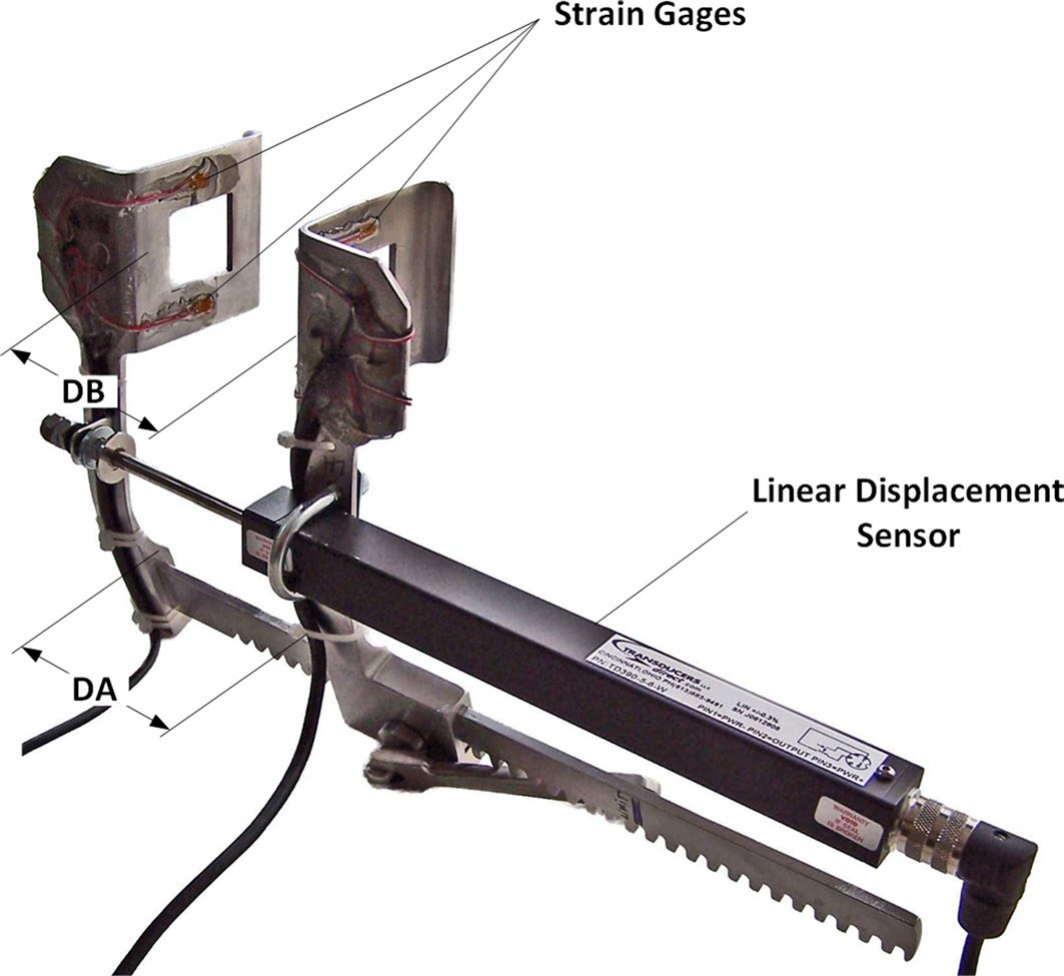
Instrumented Finochietto retractor for quantifying retraction in animal studies. Each blade has four strain gages mounted in a full-bridge configuration, maximizing force measurement sensitivity while compensating for temperature effects. Linear displacement sensor measures distance between retractor arms at their bases (DA), while the distance between retractor blades, DB, is measured using a modified draftsman’s caliper (not shown)

Static deflection tests included qualitative assessments of deflection modes and quantitative measurements of load vs. displacement. In the qualitative tests, deformations of the retractor components were observed, while the blades were loaded (to approximately 980 N using a noncompliant heavy cord between the blades), resulting in a fixed DB. For the quantitative tests, retractor forces and displacements (DA) were continually monitored, while the retractor was opened against rigid rods that imposed a fixed DB.

### Animal studies

Animal studies were conducted using female pigs (American Yorkshire, 50–55 kg) at the College of Veterinary Medicine, North Carolina State University (Raleigh, NC). All procedures were performed under protocols approved by the University’s Animal Care Committee. Thoracotomies were performed either on anesthetized animals (seven) or on cadavers (17). For the latter, the animal was euthanized with pentobarbital (FatalPlus, Vortech, MI) and the procedures were performed within 1 h of euthanasia. For the live surgeries, all pigs were anesthetized with Isoflurane (IsoFlo Abbott, Canada).

### Surgical procedure

Non-muscle sparing thoracotomies were performed in the fifth or sixth intercostal spaces. Briefly, a 22-cm skin incision (measured via ruler) was made. This large skin incision was used to eliminate the contributions of skin elasticity to force measurements, as pigs have much thicker and denser skin than humans. The first layer of muscle (*latissimus dorsi*) was incised and hemostasis was performed. Then, the second layer of muscles (*serratus ventralis*) was incised. Next, a 12–14 cm incision was made through the intercostal muscles midway between the ribs, and the instrumented retractor was inserted midway along the length of the incision. Retraction proceeded from the 0-s mark at a predetermined rate of approximately one half rotation of the crank (referred to here as a “click”) every 10 s.

During each thoracotomy, the retraction force and displacement (DA) were continually monitored. Immediately following the conclusion of each retraction, blade displacement (DB) was measured with the retractor fully loaded in situ. Procedures were videotaped for re-examination of surgical motions, retractor kinematics, and for the sound of cracking ribs, which has a distinctive timbre.

The acquired data were examined post-operatively to identify “ruptures”—defined as discrete events in the force traces, indicating that a component (e.g., a ligament or a rib) had broken or failed. Two criteria were used to identify ruptures: (1) a large, sudden decline in force (> 15 N in less than 0.25 s) or (2) a “saturation” of the force between successive clicks (force increased less than 10 N from the previous click).

## Results

### Kinematic analysis of the crank mechanism

The Finochietto crank mechanism is a rack-and-pinion drive using a two-post-pinion (Fig. 2a–c). This drive is “self-retaining”: the handle locks automatically under load at each click, so the retractor holds position without a second lock mechanism. Locking occurs whenever the line connecting the centers of the two posts is parallel to the direction of drive. Starting at a zero position (0°) in which a line drawn between the two centers of the posts of the pinion is aligned parallel to the rack, rotation of the crank begins to drive the rack. At a rotation of 90°, speed is maximal (Fig. 3). At each half rotation (every 0° and 180°), the speed goes to zero (Fig. 3). In fact, the direction of motion reverses slightly. This back drive is the basis of self-retaining action; after the back drive, the blades must be separated slightly to rotate the crank in either direction. However, the loaded tissues are forcing the blades together, thereby locking the rack-and-pinion in position. This mechanism, thus, produces a retraction speed that is highly non-linear, including brief moments of backward motion (Fig. 3).

**Fig. 2:**
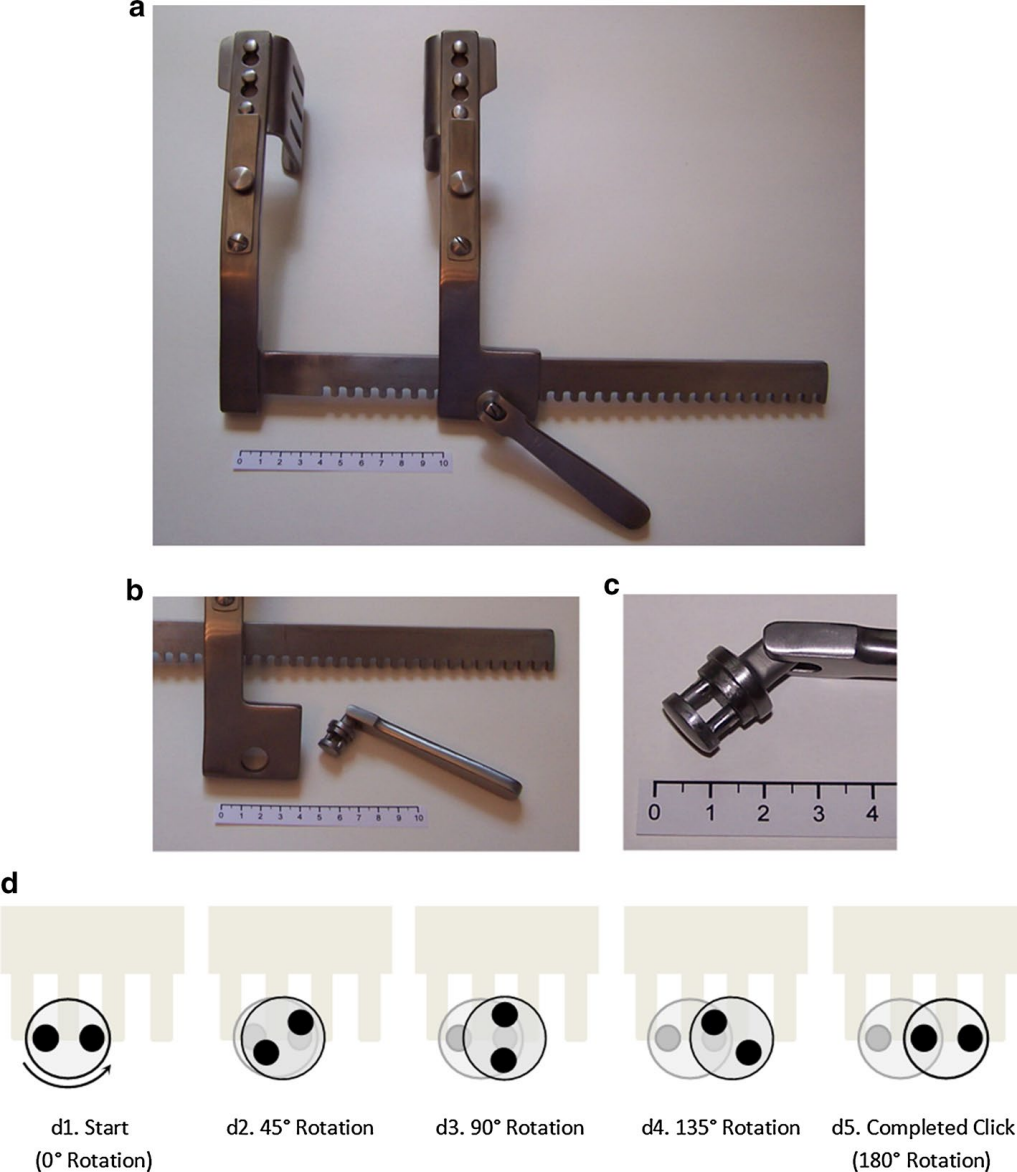
Actuation kinematics of a Finochietto-style retractor (rule = 10 cm). **a** Assembled retractor. **b** Rack-and-pinion mechanism for actuating retractor. **c** Close-up of two-pin gear of rack-and-pinion. **d1**–**d5** Diagram showing sequence of rotation for the two-pin gear and how it generates motion. **d1**: Start position; **d2**: 45° rotation—the right-hand pin slips further into the teeth of the drive as the left-hand pin moves out of the teeth; **d3** 90° rotation—the gear has translated a distance equaling half the distance separating the two pins; **d4**: 135° rotation; **d5**: 180° rotation (half turn completed)—the blade has moved a distance equal to the spacing of the gear teeth (5–9 mm for most retractors). At 0° and at 180°, the retractor locks

**Fig. 3:**
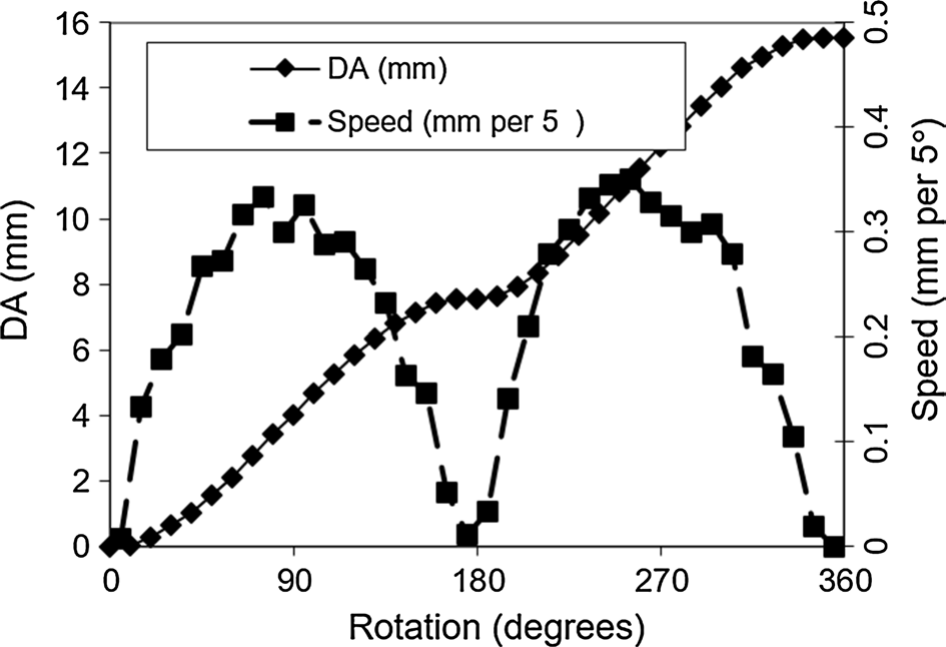
Speed and displacement of the blade as a function of rotation of the handle: result of two clicks (one complete rotation) of the handle. The blade speed is zero at the start and end of each click (0° and 180° and 360°) and maximal at each mid-click (90° and 270°)

### Static deflection tests

Three major deflection modes were observed under load (Fig. 4a–c): (1) the rack bending out of its initial unloaded plane (*xy*), with the center of the rack deflecting (*d*) toward the operator; (2) the distal ends of the arms (and so the distal edges of the blades) deflecting torsionally (about the *z*-axis, *α*), and (3) the arms of the retractor deflecting torsionally (about the *y*-axis, *β*), causing the blades to come together, such that the distal edges of the blades are closer than the proximal edges.

**Fig. 4:**
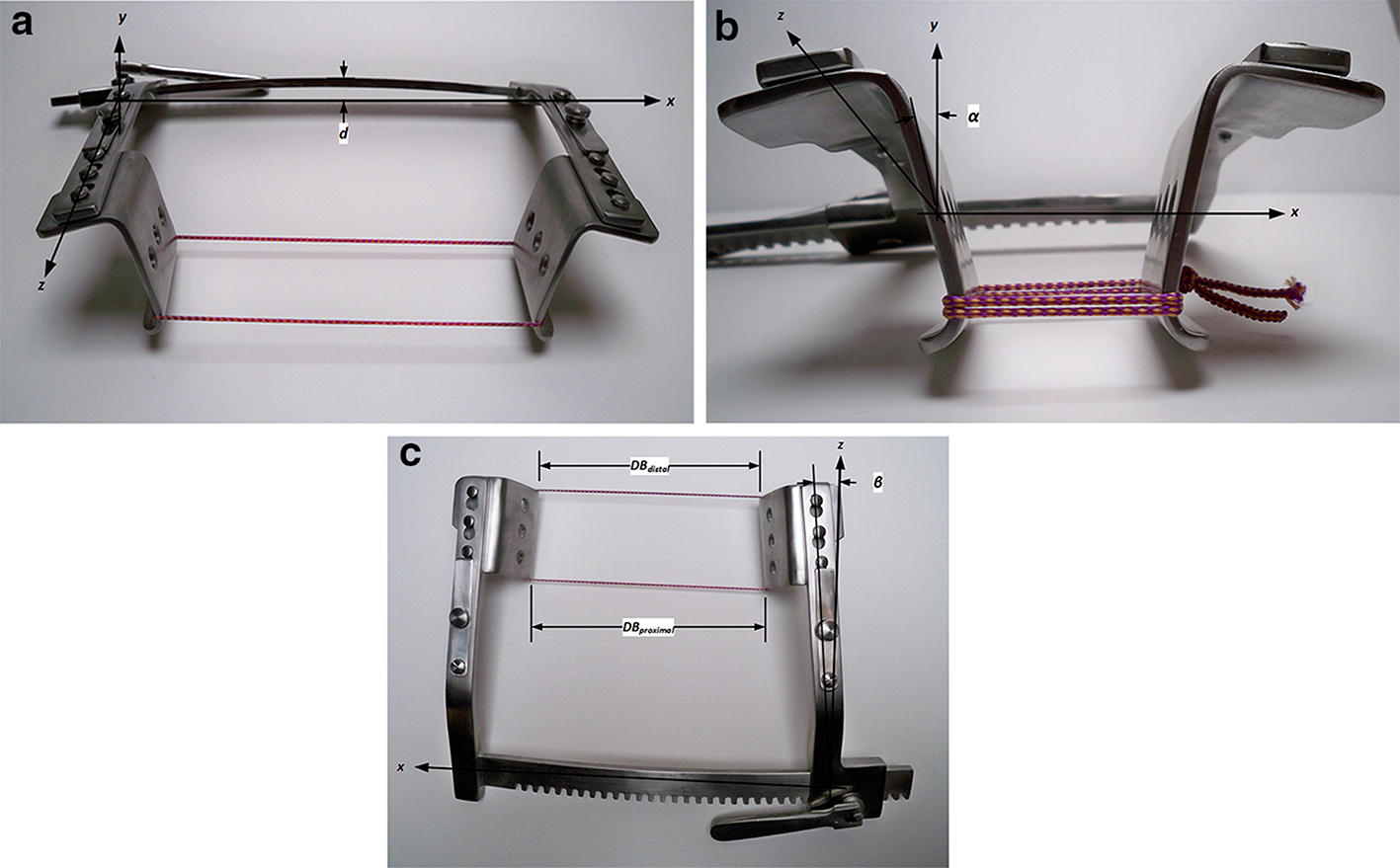
Deflection of a Burford retractor under loading (approximately 1000 N): **a** Retractor opened wide to illustrate the rack bending out of its initial unloaded plane (*xy*), with the center of the rack deflecting (*d*) toward the operator; **b** the distal ends of the arms (and so the distal edges of the blades) deflecting torsionally (about the *z*-axis, *α*); **c** the arms of the retractor deflecting torsionally (about the *y*-axis, *β*), causing the blades to come together, such that the distal edges of the blades are closer than the proximal edges

The consequences of these deformations are: (1) the combined bending and twisting of the arms illustrated in Fig. 4b, in combination with the forces exerted by the blades on the tissue, creates resultant vertical (*y*-direction) forces that drive the blades upward and out of the incision until the hooked bottoms of the blades engage tissue. (2) The combined bending and twisting of the arms illustrated in Fig. 4c causes the distal edges of the blades (furthest from the rack) to deflect away from the incision and, thus, concentrate force on the proximal edges.

### Retraction during thoracotomy

Twenty-four surgical retractions were recorded. We observed similar results in both cadaver and live animal surgeries; therefore, all retractions were pooled for data analysis. Retractions ranged from 50 to 112 mm (DA) during 30 to 370 s. Maximum retraction force ranged from 118 to 470 N (12–50 kgf, Fig. 5a, b).

**Fig. 5:**
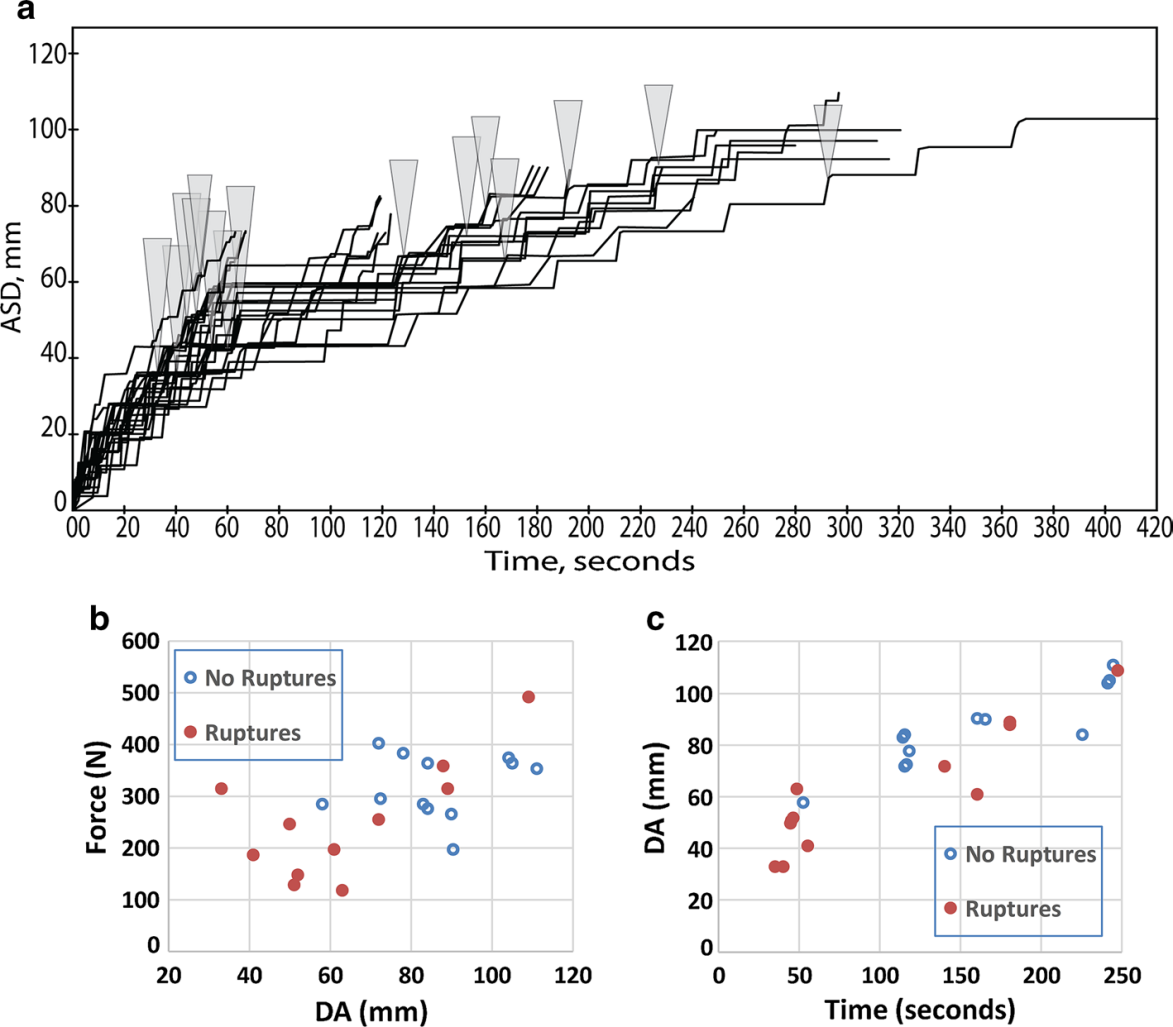
Force and displacement for 24 thoracotomies. **a** Arm displacement (DA) time series, with occurrence of initial rupture designated by gray triangles. **b** Force vs. displacement plots, with procedures resulting in rupture indicated. **c** Final displacements (DA) vs. time durations, with procedures resulting in rupture indicated

All retractions were analyzed for ruptures; which indicate failure in one or more tissue components, and thus, subsequent measurements are not appropriate. For retractions without ruptures, we used the maximum force achieved during retraction and the displacement (DA) corresponding to that force, and for retractions with ruptures, we used the force and displacement at rupture. Twelve retractions were scored as having ruptures for a total of 15 ruptures (13 determined by Criterion 1 and two by Criterion 2). Rib fracture was positively identified by inspection in three retractions, but may have occurred more frequently, as explained later. Twelve retractions were scored as not having ruptures. There were no ruptures below 30 mm displacement (Fig. 5) or below 122.5 N (data not shown). Otherwise, there were no apparent relations between the duration and distance of retraction with ruptures. Some ruptures occurred at displacements as small as 32 mm (with as little as 122.5 N), while some retractions proceeded to displacements of 112 mm (382 N) with no apparent ruptures.

Retraction proceeded in steps, one for each click (Fig. 6). While retraction spanned 240 s, deformation actually occurred in 14–15 steps each spanning approximately 2 s, so all deformation occurred over only about 30 s total. Thus, while the average retraction rate (i.e., 112 mm/240 s = 0.47 mm/s) was slow, the instantaneous retraction rates were much higher (around 4 mm/s). After the first two or three clicks, each click increased force by 40–70 N over the previous values, becoming larger as retraction proceeded. Each click was followed by a period of force relaxation (Fig. 6b). Audible rib fractures were heard for the first and third ruptures, and at the conclusion of retraction, both ribs were visibly fractured at the proximal edge of each retractor blade. Note that the first rib fracture (first rupture) and the second rib fracture (third rupture) occurred after the click had completed.

**Fig. 6:**
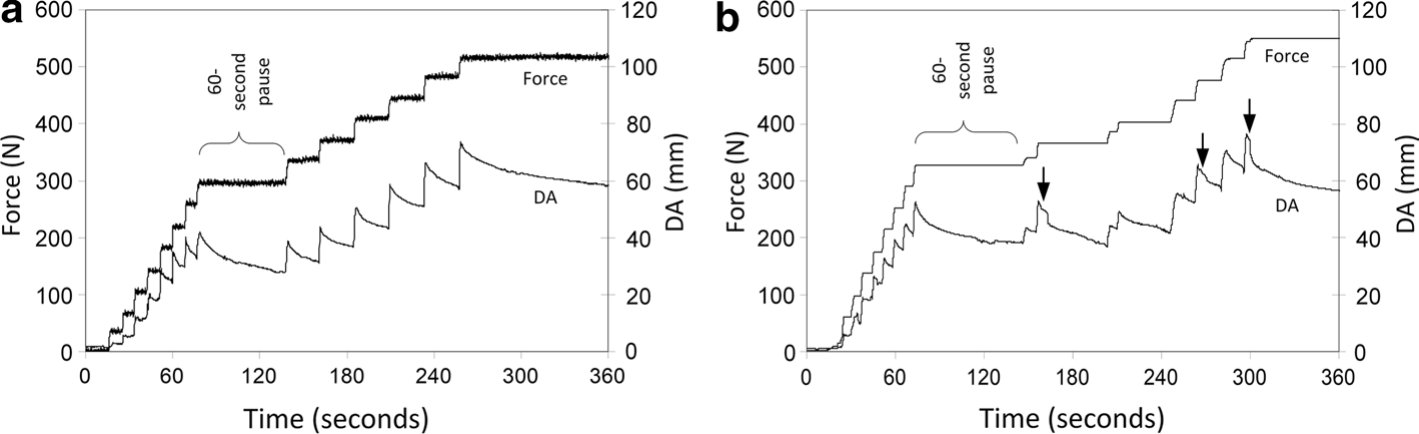
Force and displacement for two thoracotomies. **a** No ruptures evident. Retraction to DA = 112 mm over 240 s. Retraction proceeded as eight clicks in the first 60 s, followed by a 60 s pause, and completed with a final six clicks. Each click is evident as a step increase in both traces. After each click, the force decreases due to force relaxation of the viscoelastic tissues. Maximum force was 372 N (38 kgf) at the end of the final click. DB at end of the final click was only 93 mm (19 mm less than DA). **b** Three ruptures evident (marked by arrows). Retraction to DA = 115 mm over 240 s. Retraction proceeded as nine clicks in the first 60 s, followed by a 60 s pause, and completed with a final clicks. Maximum force was 372 N (38 kgf) at the end of the final click and would have been higher if the ribs had not fractured. DB at end of the final click was only 93 mm (21 mm less than DA)

Thoracic tissues are viscoelastic, as evidenced by force relaxation: the significant reductions in force that occur after the tissue has deformed (Figs. 6, 7). Deformation of viscoelastic tissues at more rapid rates (e.g., faster retractions) requires larger forces. To confirm this behavior, we pooled all retractions that reached 70 mm displacement without ruptures (*n* = 15) and analyzed force and retraction rates at DA = 70 mm. These showed a weak positive correlation between retraction rate (*x*) and force (*y*) (data not shown, *x* = 31.84*y* + 8.03, *R*^2^ = 0.138, *p* = 0.061; rates ranging from 0.36 to 1.13 mm/s, forces ranging from 149 to 370 N).

**Fig. 7:**
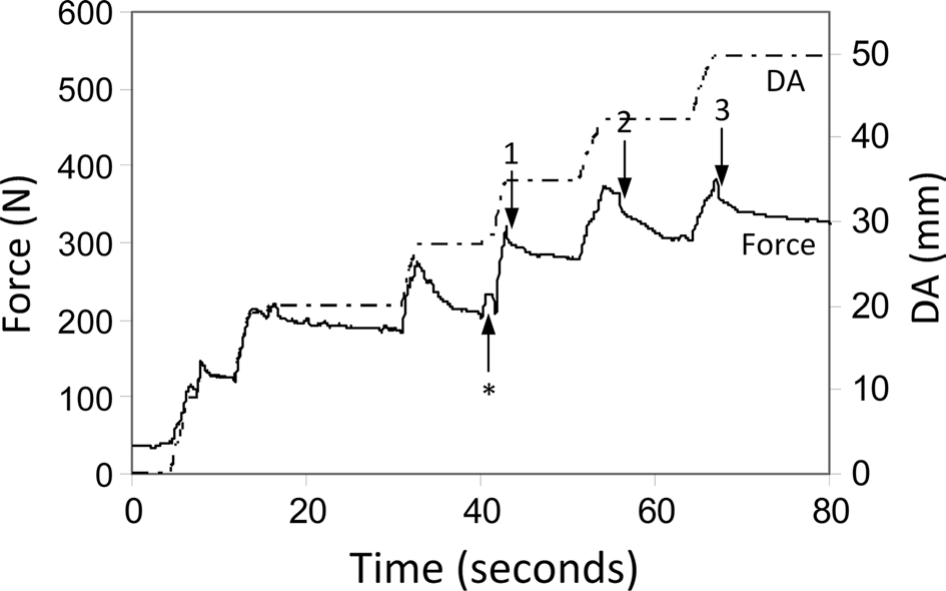
Force and displacement for a thoracotomy with three ruptures evident (marked by arrows). Retraction to DA = 50 mm over 60 s. Retraction proceeded as six clicks. Surgeon changed the position of his hand at the point marked with an asterisk. Three audible rib fractures were heard (marked with numbered arrows). The force change when the surgeon changed the position of his hand is similar to that seen for the rib fractures

In addition, when all 24 retractions were examined at full retraction, there were ruptures in all but one of the retractions with average retraction rate equal to or exceeding 0.75 mm/s (*n* = 7). This is equivalent to a pace of about one click every 10 s, indicating that retractions exceeding one click every 10 s almost always resulted in rupture. When considering cases that resulted in ruptures, retractions at higher rates ruptured at lower forces (*R*^2^ = 0.364, *p* = 0.038). This suggests that thoracic tissues are more fragile when faster retractions are used. Almost all materials, viscoelastic or not, require higher forces for larger deformations. A significant relationship between retraction force (*y*) and displacement (DA, *x*) was found (*y *= 0.223*x *+ 12.9, *R*^2^ = 0.292, *p* = 0.0064). However, the ratio of force to DA (effectively the “stiffness” of the retracted tissue) varied widely between individual retractions.

## Discussion

This study demonstrates that thoracotomy is a remarkably forceful procedure, requiring forces ranging from 165 to 470 N (i.e., 33% to nearly 100% of the animal’s weight). The forces that we measured for pigs are similar to those reported for thoracotomy in sheep, sternotomy on human cadavers (Aigner) and higher than for sternotomy in sheep by Bolotin et al. [25–27]. We noted that large changes in force could be created by small actions of the surgeon (like feeling along the margin of the incision with a finger or attempting to stabilize the retractor before turning the crank), and these operator-induced changes can obscure changes in force arising from tissue rupture, such as a rib fracture (Fig. 7).

We also observed force relaxation as reported by Bolotin et al. in sheep [25–27]; this was evident every time retraction paused. During such pauses, force decreased by 30–40% over 1 min (see Fig. 4), and at the conclusion of retraction, force dropped by 10–20% in the first minute, similar to that observed by Bolotin et al. Interestingly, we usually observed a 50% force reduction over 1 h (approximating the duration of a surgical procedure), indicating that thoracic tissues can dramatically relax over time. These findings are in agreement with studies done by Saggio et al. [24], who used instrumented Finochietto retractors and documented similar sternal forces using a similar retraction rate (5 mm/s vs. 4 mm/s in our study) and maximum opening gap.

We utilized dual criteria to quantify the incidence of rib fractures because of the difficulties in reliably evidencing fractures by either singular method. The relationship between “ruptures”, as scored from the force traces, and fractures of anatomical elements is not obvious. Characteristic cracks (like the snapping of a tree branch) during surgery were always accompanied by rapid drops in the force trace of 15–50 N (Criterion 1); however, large drops in the force trace sometimes occurred when there was no audible snap. Failure of the force peak at one click to exceed the peak of the previous click (Criterion 2) was usually accompanied by a series of smaller snapping sounds. In addition, rib fractures were not always evident when the ribs were examined after surgery, even when a large snap was heard. In the previous studies performed with aims that differ from this study (data not shown), we regularly observed rib fractures that were not evident until the ribs were completely dissected from other tissues. Thus, there may be rib fractures, possibly microfractures or occult fractures, which are not detected during surgery by simply inspecting the incision, mirroring sternotomy side effects, where rib fractures are very hard to detect, even by radiographs [28].

Some results agreed with intuition, especially in light of the viscoelasticity of thoracic tissues:

1. There were no ruptures at smaller retraction distances (DA < 32 mm) and at lower forces (< 125 N).
2. The highest retraction rates (> 0.75 mm/s, *n* = 7) almost always produced ruptures.

However, some results conflicted with intuition:

1. Larger retractions (larger DA) and larger forces did not always result in more tissue ruptures. Ruptures occurred over a wide range of retraction forces, from 125 to nearly 500 N, and many retractions showed no obvious ruptures despite achieving high forces (up to 400 N).
2. Force increased with increasing retraction distance within a given procedure, but the relationship varied widely between different retractions, despite all animals being of similar size and age.

We identified the following aspects of Finochietto-style retractors that could potentially be addressed in future retractor designs to reduce tissue trauma:

1. Smooth manual velocity control is not feasible owing to the non-linear, oscillating relationship between crank rotation and arm motion.
2. Force sensing by touch is difficult due to stiction in the drive mechanism and to the non-linear relationship between crank rotation and arm motion.
3. Fine control is most difficult when it is most critical: the crank is hardest to turn when tissues are most stressed (and forces are highest) at fullest retraction.
4. Motion is quantized to steps equaling the tooth spacing of the rack (steps of 8 mm are common on medium-to-large retractors, which is 5–10% of a typical retraction). Therefore, fine adjustment of the opening, such as when the tissues are heavily loaded, is not possible.
5. The retractor behaves like a spring that continues to force open the incision after crank rotation has ceased. We observed increases of up to 15 mm for an 85 mm opening (nearly a 20% increase) after ‘finishing’ a retraction. This continued, unintended retraction causes additional tissue damage: 39% of ruptures occurred after a click was completed. While this may seem contrary to classical engineering failure theories (e.g., the distortion energy criterion or the maximum shear stress criterion) that relate failure to stress rather than strain, this continued tissue relaxation appears to transfer load to less mobile tissue, resulting in failure.
6. The blade edges on most Finochietto-style retractors cause stress concentrations that increase the probability of rupture; deformation of the retractor under load greatly concentrates this stress on the proximal edge.

These results lead to several recommendations for decreasing tissue trauma during retraction with a Finochietto retractor:

1. To avoid force concentration, pad the blades of the retractor, especially the proximal edges of the blades, and do not place the proximal edges of the blades closer to the end of the incision that is nearest the rack.
2. Open the first 30 mm (~ 4 clicks, smaller distances for smaller patients) more quickly, because forces are lower at the beginning and less likely to cause fractures, and rapidly loading the tissue during this portion of retraction accelerates subsequent force relaxation. After the first 30 mm, open more slowly, being especially careful not to retract faster than one click every 10 s. Thus, completing the final 80 mm of a 110 mm retraction should require no less than 100 s; slower is certainly better, because it allows greater force relaxation.
3. Long pauses (e.g., 1–2 min) are helpful, as they permit force relaxation, but distributing more, smaller pauses more evenly throughout retraction (e.g., 20–30 s of pausing after each click), especially after each of the last few clicks, permits larger total force relaxation, decreasing maximum force of retraction.
4. For later clicks when tissues are more heavily loaded, turn the crank slowly, especially at the 90° position, and pause for 10 or more seconds at 90° (by holding the hand crank at mid-step) to permit force relaxation.
5. Stop one click short of the desired retraction if it is acceptable to let force relaxation/creep of the tissue open further over the next 5 min.
6. Avoid unnecessary motions of the retractor after the first four clicks. Even small adjustments produce large changes in the forces applied to the tissue (due to strain-induced changes in mechanical advantage between the retractor blade and tissue). In addition, carefully stabilize the retractor with one hand while rotating the crank with the other hand as smoothly as possible, and avoid pushing, lifting, rotating, or otherwise moving the retractor after it is loaded.

The main limitation of this study is the experimental model. We acknowledge that the surgical incision performed here does not mimic exactly the incision used in a clinical setting and that this difference may have impacted our results. However, informal discussions with numerous thoracic surgeons revealed that there is no “standard” thoracotomy approach. Furthermore, although widely used in biomedical research, the pig has significant differences from humans regarding chest anatomy (e.g., flattening of the chest wall in the orthogonal plan, different placements of muscle attachments, strength of the skin, etc.) that necessitated our incision modifications. In addition, the aforementioned difficulties in detecting all rib fractures, particularly microfractures and occult fractures, were limiting; future work will focus on the development of more advanced sensing and detection strategies, and the development of automatically controlled retraction prototypes that limits the magnitudes and rates of retraction forces.

Despite these limitations, we believe that this study delivers sound information on the mechanics of self-retaining thoracic retractors. More extensive analyses of the biomechanics of retraction are needed to enable newer designs that reduce trauma while still achieving surgical access.
